# Maternal singing sustains preterm hospitalized newborns’ autonomic nervous system maturation: an RCT

**DOI:** 10.1038/s41390-023-02932-4

**Published:** 2023-12-06

**Authors:** Manuela Filippa, Mimma Nardelli, Alessandra Sansavini, Sara Meloni, Odoardo Picciolini, Clara Lunardi, Alessandra Cecchi, Luigi Corvaglia, Didier Grandjean, Enzo Pasquale Scilingo, Elisa Della Casa, Alberto Berardi, EVC Group, Arianna Aceti, Luca Bedetti, Natascia Bertoncelli, Giovanna Lucco, Michele Luzzati, Luca Ori, Chiara Petrolini, Mariagrazia Zuccarini, Fabrizio Ferrari

**Affiliations:** 1 Division of Development and Growth, Department of Pediatrics, University of Geneva, Geneva, Switzerland; 2 Department of Psychology and Educational Sciences, University of Geneva, 24, rue General Dufour, 1211 Geneva, Switzerland; 3 Bioengineering and Robotics Research Centre E. Piaggio and Dipartimento di Ingegneria dell’Informazione, University of Pisa, 56122 Pisa, Italy; 4 Department of Psychology “Renzo Canestrari”, University of Bologna, Viale Berti Pichat 5, 40127 Bologna, Italy; 5 Pediatric Physical Medicine & Rehabilitation Unit, IRCCS Ca’ Granda Ospedale Maggiore Policlinico, Via Francesco Sforza, 35, 20122 Milan, Italy; 6 Department of Neurosciences, Psychology, Drug Research and Child Health, Careggi University Hospital of Florence, Florence, Italy; 7 Division of Neonatology, Careggi University Hospital, University of Florence School of Medicine, Florence, Italy; 8 Neonatal Intensive Care Unit, IRCCS AOU Bologna, Via Massarenti 9, 40138 Bologna, Italy; 9 Women’s and Children’s Health Department, Neonatal Intensive Care Unit, University Hospital of Modena, Modena, Italy; 10 Department of Medical and Surgical Sciences of Mother, Children and Adults, University Hospital of Modena, Modena, Italy

## Abstract

**Background:**

Premature birth is known to affect the newborn’s autonomic nervous system (ANS) maturation, with potential short and long-term impact on their neurobehavioral development. The purpose of the study was to investigate the effects of maternal directed singing and speaking on the preterm infants’ autonomic nervous system (ANS) maturation as measured by the heart rate variability (HRV) parameters.

**Methods:**

In this multi-center randomized clinical trial, 30 stable preterm infants (m = 29,6 weeks of gestational age), without any abnormalities were randomized into an intervention (16) or a control group (14). HRV was measured weekly, for a total of 80 recordings during hospitalization, as well as before and after each session of singing or speaking.

**Results:**

The intervention group showed a significant increase of the percentage value of HRV power in the high frequency range when compared to the control group (*p* = 0.044). More specifically, the maternal singing significantly increased the high frequency power and decreased the low/high frequency power ratio (*p* = 0.037).

**Conclusions:**

The preterm infant’s vagal activity significantly increased in the intervention group, potentially enhancing their ANS maturation. The effect is specifically evidenced in the singing condition.

**Impact:**

- Maternal singing affects the autonomic nervous system maturation of preterm hospitalized newborns in the NICU.
- No previous studies investigated how early vocal parental intervention can affect preterm infants developement, throught their autonomic nervous system maturation.
- Early Vocal Contact as an early intervention involving parents has a positive impact on preterm infant’s development and it can be easily implemented in the care of preterm infants.

**Trial registration:**

NCT04759573, retrospectively registered, 17 February 2021.

## Introduction

The variability of the heart rate is suggested as an important, and non-invasive, instrument for assessing the autonomic nervous system (ANS) functioning and development in the neonatal period.^1,2^ The newborn’s cardiovascular system is in fact mainly controlled, and dependent on the maturation of the ANS.^3^

If heart rate measures are currently used in clinical practice, in conjunction with other physiological measures, for detecting critical events during hospitalization (e.g., bradycardias, tachicardias, apnoeas), HRV is not a routine assessment of neonatal wellbeing for populations at risk, such as premature newborns.

The Task Force of Cardiology identifies the standards of measurement for HRV and defines its physiological interpretation.^4^ Each identified frequency range, the low (LF) and high frequency (HF), corresponds to the involvement of the sympathetic or parasympathetic system. LF frequencies vary from 0.04 to 0.15 Hz and correspond to the involvement of the sympathetic system predominantly and the parasympathetic system secondarily. HFs in newborns range from 0.15 to 2 Hz and are mostly controlled by the parasympathetic nervous system. Normalized indices (LFnu, HFnu) are used, and together with the LF/HF ratio they assess sympathetic modulation and autonomic equilibrium in the neonatal period.

Using a machine learning model, HRV data from full-term and preterm infants are promisingly utilized as a reliable measure to predict infant’s functional maturational age.^5^ However, if normative data for establishing newborns’ physiological autonomic maturation profiles have been collected for term newborns,^6^ the preterm infants’ HRV developmental trajectories from birth to term equivalent age are still under investigated.

Preterm newborns present an atypical characterization of the HRV features, when compared to full-terms.^7,8^ As the complete maturation of the autonomic control system is expected to be achieved around 37 weeks of gestational age *in utero*, preterms are born with an incomplete maturation process, which affects in particular the maturation of their parasympathetic system.^9,10^

At term equivalent age, the degree of prematurity predicts lower parasympathetic activity, and HF components of HRV are found to be significantly lower in the preterm than in the full-term group.^1,11^ Full-terms show higher values of beat-to-beat variability compared to a high-risk preterms, but the overmentioned HRV disfunction also affects late preterm infants, who usually show less risk factors than their pairs born before 32 weeks of gestational age.^12^

As preterm infants age, there is a large increase in HRV parameters, especially in the HF, which is interpreted as a main indicator of parasympathetic maturation.^13^ In turn, the progressive activation of the parasympathetic tone reflects the gradual maturation of the newborn’s ANS. The development of the newborn brain during the earliest stages of the neonatal period ensures that higher cortical processes integrate autonomic control, and this process results in a predominant parasympathetic activity in overall autonomic maturation during the first 2 years of life.^6^

### Social contact modulates HRV in neonates and infants

Infant’s HRV is modulated both by endogenous and exogenous events, making a link between the establishment of an inner equilibrium and the response to external social and perceptual stimuli.^14^ This balance permits, for instance, the emergence of a contingent response to external salient sensory stimuli, which serves as the foundation for subsequent social experiences.

In fetuses, the cardiac system can adjust its activation in response to external—in this case maternal—stimulation.^15^ Authors acquire the heartbeat time series of mothers and fetuses under different breathing conditions and fetal–maternal synchronization is identified based on synchrograms. HRV can thus provide a physiological basis for detecting the fetuses and newborn’s abilities to synchronize with caregivers and to respond to social cues. During infancy, the cardiac interaction between mothers and their babies is demonstrated, and, in particular, the changes in maternal HRV modulated the Low frequency (LF) components of the HRV both in younger (3–5 months) and older (6–8 months) infants.^16^

In the newborn population several studies establish the modulatory effect of an early parental – tactile, vocal or multimodal – intervention on preterm infant’s HRV.

Kangaroo-care increased maturation in the preterm infants’ vagal activity, increasing in particular their parasympathetic activity,^17^ active parental implication in nurturing interaction with preterm newborns enhance their autonomic regulation, with long-term effects on the dyad,^18^ and dynamic touch impacts on the autonomic nervous system response, inducing a more balanced activity in preterm infants, with potential autoregulatory functions.^19^

### Maternal voice perception in preterm infants

The mother’s voice represents a fundamental social stimulus for newborns, who can discriminate and prefer it over stranger voices.^20,21^ Term newborns tend to process the maternal speech at a preattentional level, preferentially in the left temporal lobe, before activating central right areas, probably evoking a sensorimotor response.^22^ The possible presence of an innate auditory-articulatory loop is also confirmed by a recent fMRI study, comparing singing voice versus instrumental melody perception in newborns (Loukas et al., in press).

Preterm infants, at term equivalent age, show an atypical voice processing when compared to term newborns: ERP studies demonstrate that preterm newborns tend to process similarly mother’s and stranger’s voices,^23^ they recruit additional cortical regions involved for processing voices as evidenced with in fMRI technique^24^ and in multiple frequency bands, only full-term newborns show an increased activity for the mother’s voice, whereas preterms show significant activation for stranger speech.^25^

As brain networks selectively engaged in children by their mother’s voice predict children’s social communication abilities,^26^ early interventions in the NICU should seek to reestablish an optimal bioecological niche where preterms can develop preference and salient sensitivity to their mother’s voice.

### Maternal singing and speech in the NICU

The results of several systematic reviews conducted in the last years confirm that mother’s voice increases preterm infant’s physiological stability, has beneficial impacts on the infant’s nutritional domain and decreases maternal anxiety.^27–29^ Parental singing sustained by a music therapist, during skin to skin contact enhances neural discrimination of sound changes^30^ and is correlated with larger neural responses to deviant sounds^31^ in preterm newborns. Maternal speech and singing during a painful procedure decrease pain and increase preterm infant’s oxytocin level, marginally significant for singing,^32^ and, concomitantly, increase oxytocin in mothers with a decrease in their anxiety levels.^33^

More specifically, regarding the impact of maternal voice on the ANS regulation, preliminary results demonstrate that the maternal singing modulates the preterm infant’s HRV in the short term, with increased values during the maternal singing.^34^ These preliminary results confirm thus the potentiality of early vocal contact as an early intervention for preemies in the NICU.^35^

To which extend these immediate modulations of the ANS as a result of vocal contact can favorably impact the ANS maturation is still unknown.

Our main aim is to test the modulatory effects of live maternal singing and speech on the ANS maturation in the preterm hospitalized infants’ population.

This study is a part of a larger study investigating the short and long-term effects of EVC on preterm infants.^34^

## Materials and methods

### Participants

The present data are based on a subsample of the larger cohort of the Early Vocal Contact Project,^36^ in which 60 preterm newborns were recruited from four hospital centers, Modena and Florence University Hospitals, Milan Mangiagalli Policlinico and Bologna University in collaboration with their respective IRCCS Hospitals. The study was approved independently by each center’s Ethical Committe (P.0006292/18, for details see Institutional Review Board Statement).

A final sample of 30 preterm infants, between 25 and 33 weeks of Gestational Age (GA) at birth, had complete ECG recordings and were included in the present analysis. Twelve neonates were excluded for incomplete data or too low quality of the acquired cardiovascular signals, and 18 preterm neonates were excluded because the data were collected using a system that did not output raw PPG data and later proved to be incompatible with the data collected in the other centers during analysis.

In the present RCT study trial, neonates were randomly assigned to intervention (16) or control groups (14) with a randomization process stratified by sex and GA. The randomisation sequence, unique for the four centres, is led by the Statistical Office of the University of Modena and Reggio Emilia. Characteristics of the involved population are summarised in Table 1.

**Table 1:** Participant’s characteristics.

	Intervention	Control
Infants’ characteristics	*N* = 16	*N* = 14
Gestational age at birth (weeks), mean *(SD)*	29,66 (2, 27)	30 (1, 67)
Birthweight (g), mean *(SD)*	1201 (328, 87)	1228, 18 (236, 48)
Apgar score at 5 min, mean *(SD)*	6, 75 (1, 26)	6, 82 (0.87)
Sex (%)	Female (50%)	Female (64, 28%)
Mother’s age (years), *mean (SD)*	34 (1, 89)	35, 4 (7, 35)
Gestational age at test (weeks), *mean (SD)*	34, 91 (1, 55)	34, 27 (0, 83)
Weight at test (weeks), *mean (SD)*	1811, 62 (256, 01)	1652 (287, 03)
Post Natal Age at test (days), *mean (SD)*	36,56 (19, 14)	32, 36 (16, 38)
*N* of recordings	45	42

Families were enrolled in each center after informed consent was obtained through recruitment procedure. Preterm newborns were included if their GA was between 25 + 0 and 33 weeks at birth and if birth conditions - Apgar score, birth weight, birth cranial circumference were appropriate for GA. Newborns were excluded if they presented sepsis or congenital and genetic anomalies. Newborns requiring high flow oxygen support at test were not included. Finally, only mothers without declared depressive symptoms or without history of drug abuse were enrolled in the study.

### Intervention

The intervention consisted in asking mothers to speak and sing to their newborns, for 20 min, 10 for speaking and 10 for singing, and the order of two conditions was reversed in the following day. The intervention occurred three times per week for two weeks, midway between the feeding cycles, more than one hour after the feed, and more than four hours after the last medical examination. The newborn’s state has been checked by a trained nurse, and the intervention did not begin when newborns were in deep sleep or crying states. Mothers were free to choose the content of their speech and songs and background noise measures were taken with a sound level meter (Voltcraft Phonometer SL-10), before each intervention, in the NICU room and inside the incubator, 10 cms from the newborns’ heads, to ensure that the mother’s voice was audible, when it exceeded the background noise of 10 dBA (for details see (Kuhn, 2012 #433)).

Mothers were encouraged to talk in their native language and to sing familiar songs, while observing their infant’s reactions, without touching the baby.

An active control group provided comparative data, and mothers were instructed to observe their infant’s behavior for the same amount of time and record their observations using a coding grid established for parents based on the Neonatal Behavioral Assessment Scale. During this time, mothers were encouraged to remain close to the incubator but to refrain from speaking or singing to their newborns.

### Main outcome and outcome measures

The main aim of the study was to measure the infant’s HRV in the different sessions of the interventions and control period. At every session the photoplethysmography (PPG) signal was acquired from a sensor placed on the feet of each newborn during a baseline period (20 min before the intervention, in absence of the mother), during the vocal intervention (20 min) and after it (20 min after the intervention). No additional measurement was acquired to the routine physiological acquisitions, but the PPG data were automatically recorded by the IxTrend Software (Ixellence, Germany). The same procedure was carried out in the control group. Each subject experienced a minimum of one recording session of the whole experimental protocol and a maximum of six repetitions of the same experiment, with a distance of 3 days between two successive repetitions. A total of 45 and 42 signal recordings were conducted for the intervention group and the control group respectively.

### Cardiovascular data analysis

After a preliminary analysis of the quality of the PPG signals acquired in the centers participating in the study, the cardiovascular signals were studied on a total of thirty subjects, divided into two groups: the intervention group (16 newborns) and the control group (14 newborns). The PPG signals were then pre-processed to remove noise and motion artifacts. For each subject, a series of pulse-to-pulse (PP) time intervals was extracted from each PPG signal in order to analyze the related heart rate variability (HRV). The details of these processing steps are described in the following paragraphs.

#### PPG signal processing

PPG signals were acquired at a sampling frequency of 125 Hz, and then filtered using a second-order 0.5–10 Hz Butterworth band pass filter. The pulse detector algorithm described in^37^ was used to identify the pulses related to heartbeats. This technique consists of a linear filtering transformation based on a FIR low-pass-differentiator filter and an adaptive thresholding technique. After the pulse-detection procedure, the PP time interval series was derived, and the smoothness priors’ approach was applied as detrending method.^38^ Further possible artifacts were corrected using Kubios HRV software.^39^ We compare the cardiovascular dynamics in time windows lasting five minutes taken from each experimental condition. Considering the intervention group, we used four-time windows: the last five minutes of the first baseline period, the first five minute of the singing elicitation, the first five minutes of the speaking, and the five minutes following the vocal contact in the final baseline. Concerning the control group, considering that mothers in the control group were required to observe their infant throughout the entire session, we considered three-time windows: two five-minutes windows taken from the two baseline periods and a five-minute window taken from the twenty-minute session when the mother was observing the infant.

#### HRV feature extraction

HRV time series were derived from the PP series after a cubic spline interpolation at 4 Hz. The power density spectrum (PDS) referred to each time window was obtained by using a non-parametric estimation based on an autoregressive model.^40^ Then, we investigated the content of the PDS in the two main bandwidths for HRV analysis in the frequency domain, i.e. low frequency (LF, between 0.02 and 0.2 Hz) and high frequency (HF, between 0.2 Hz and 1.5 Hz) bands. The frequency ranges of these two bands were adapted to newborns’ cardiovascular dynamics, according to the literature.^41^ For each five-minute signal and for each of the two bandwidths we calculated the value of two features, as follows:

$$-{LF}\,{power}\,n.u.=\frac{{LF}\,{power}}{{LFpower}+{HFpower}}{and}\,{LF}\,{power} \% =\left(\frac{{LF}\,{power}}{{TOTpower}}\right)\times 100$$

$$-{HF}\,{power}\,n.u.=\frac{{HF}\,{power}}{{LFpower}+{HFpower}}{and}\,{HF}\,{power} \% =\left(\frac{{HF}\,{power}}{{TOTpower}}\right)\times 100$$

LF power n.u. and HF power n.u are the power contents in each band in normalized units (with respect to the sum of the power values in both HF and LF bandwidths), and LF power % and HF power % are the percentage values with respect to the total power of the HRV signal. In addition, we calculated the LF/HF parameter as the value of the ratio between LF power and HF power.

Given that the same subject underwent several recording sessions of the same experimental protocol (from 1 to 6), for each of the five HRV features and for each subject, the median value among all the recording sessions was computed for each experimental condition.

#### Statistical analysis

Given the non-normal distributions of the data (demonstrated with the application of the Shapiro–Wilk test), we used non-parametric statistical tests to compare the HRV features computed for the different experimental conditions. Specifically, we analyzed intra-group differences in autonomic modulation by using a Friedman test to compare the four conditions for the intervention group (initial baseline, singing, speaking, final baseline) and the three conditions for the control group (initial baseline, control condition, final baseline). When the *p*-value referred to the Friedman test was lower than the threshold of statistical significance (*p* < 0.05), a post-hoc analysis was conducted by applying the Wilcoxon signed rank test to each pair of conditions and adjusting the p-values through the Bonferroni procedure.^42^

The Mann-Whitney non-parametric test was used to compare the two groups, i.e. intervention and control groups. We applied this test to the demographic data of the two groups reported in Table 1 (age and weight at birth, Apgar 5 and Apgar 7 scores, WGA and PNA at test, and weight at test), and to the features extracted during the intervention conditions (speaking or singing) and the control condition, after the subtraction of the corresponding values computed in the first baseline period.

## Results

**Fig. 1:**
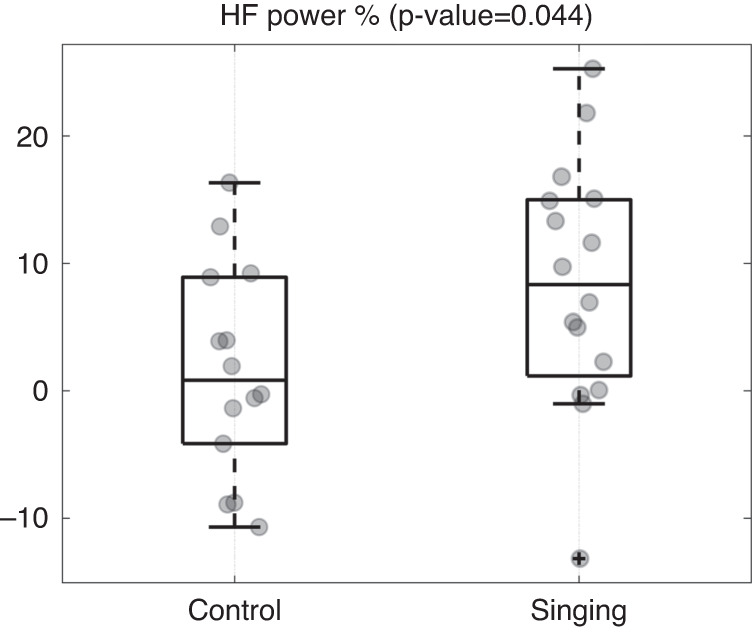
Boxplots of HF power % values computed during the singing and control conditions, after subtraction of the corresponding values obtained during the first baseline period. The *p*-value related to the Mann–Whitney non-parametric test is reported on the top. HF high frequency.

The inter-group analysis demonstrated that the modulation in vagal activity observed during the experimental protocol sessions in the intervention group was statistically different from the vagal modulation recorded in the control group. In fact, subtracting the HF power % values of the first baseline period to the values of the same feature calculated during the singing and control conditions for the two groups respectively, the non-parametric Mann–Whitney test gave a *p*-value of 0.044. Figure 1 reports on the boxplots of HF power % values of the two groups during the control and singing conditions. Finally, when we applied the same statistical test to compare the two groups according to the newborns’ demographic data, we did not find statistical differences between intervention and control subjects.

**Fig. 2:**
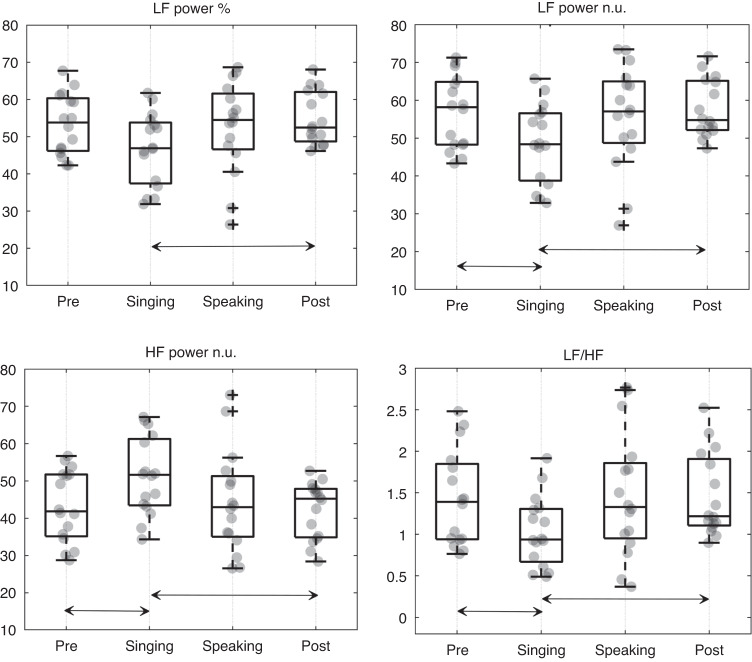
Boxplots and condition-wise statistics for LF power %, LF power n.u., HF power n.u., and LF/HF computed from the HRV signals of the intervention group. The *p*-values of the Friedman statistical test is reported next to the name of each HRV feature. The black orizontal arrow indicates a *p*-value < 0.05 of the Wilcoxon signed-rank test applied to the corresponding pair of experimental conditions, after Bonferroni correction. LF low frequency, HF high frequency, n.u.: normalized index, LF/HF low/high frequency ratio.

The intra-group analysis reported statistically significant changes in autonomic dynamics for the intervention groups. In fact, the Friedman test gave a significant p-value (lower than 0.05) for four out of five features: LF power %, LF power n.u., HF power n.u., and LF/HF. A significant decrease of the median values of LF power n.u and LF/HF was found comparing the singing condition to the first baseline period (*p*-value = 0.043 after Wilcoxon test and Bonferroni correction). Furthermore, the singing condition was demonstrated to be significantly different from the last baseline period, considering the LF power %, LF power n.u., and LF/HF features, which resulted in being lower during the intervention condition than in the last resting state (*p*-value = 0.037). A significant increase in vagal activity was reported during the singing condition. In fact, HF power n.u. values related to singing condition were higher than the corresponding values calculated from both the initial and final baseline periods (*p*-value = 0.043 and *p*-value = 0.037 respectively). Figure 2 shows the boxplots of the four features that gave statistically significant *p*-values after the Friedman test in the intra-group analysis. On the other hand, the same statistical analysis did not provide significant results for the control group, and the three conditions of the control experimental protocol were considered undistinguishable in terms of changes in infants’ autonomic dynamics.

## Discussion

In newborns, the cardiovascular system is not yet fully mature, and its development is controlled by the ANS.^3^ HRV is commonly more elevated in term newborns when compared to preterms at term equivalent age, indicating that prematurity has an impact on their parasympathetic system. In particular, the low values of the HRV are a main indicator of lack of parasympathetic maturation in preterm newborns.^13^ Our research demonstrated a beneficial effect on the developmental trajectory of parasympathetic function in preterm infants exposed for two weeks to maternal live singing during hospitalization, with increased values of HF in the intervention group.

Several longitudinal studies showed that HF presented the most marked increasing between preterms and terms.^11,13^ Even though ANS dysfunction is a trait of preterm neonates, Early Vocal Contact in our study was associated with enhanced ANS functional development.

Our results are in line with previous studies reporting improvements in preterm infants HRV,^43^ as indicated by the LF/HF ratio, after 4 weeks of massage intervention during hospitalization. Similarly, higher frequency of skin-to-skin contact with preterm infants during hospitalization is associated with higher HF during the first postnatal week and, interestingly, HF predicted earlier discharge in terms of post menstrual age.^44^

Taken together, the previously reported and the present results potentially indicate that a multimodal parental stimulation, where vocal and body contact are coherently administrated as an integral part of the individualized care, can improve the preterm infant’s neurodevelopment through the modulation of a more mature ANS. An ANS more mature, in turn, is functional to the development of higher cortical functions and sustains a correct connection with brain’s limbic structures involved in emotional state regulation.^45^ We had previously suggested that the mother’s vocal contact with her preterm infant constitutes a unique interactive context sustaining the preterm infant’s neurodevelopment.^46^ Future research aims at systematically including fathers in evidence-based trials, to evidence the similarities or the complementarity of the early parental multimodal contacts, thus including the parental proximity, both physical and psychological, as key elements for interventions in the NICU.

What might be the mechanisms by which early vocal contact affects ASN maturation, is still uncertain, but to disentangle the potential underpinnings linked to the coregulatory impact of familiar voices, we compared the immediate differential modulatory effect of the singing and speaking conditions.

Here we showed that preterm infants’ HRV is mainly modulated in the short-term by the mother’s singing voice: HF increased only during singing when compared to the pre and post conditions, and LF and LF/HF features of the HRV were significantly lower in the same comparison. The dominant activity of the LF is a frequent complication of the preterm birth, and it demonstrates a sympathetic system dominance over the parasympathetic system.^47^ In fact, during the first postnatal days, LF band activity in healthy term and preterm newborns is determined partially but significantly by sympathetic component.^48^ The simultaneous variation, increase of HF and decrease of the LF components indicates a better balance between these two factors, as well as an increase in vagal activity of the newborns. The LF/HF (low frequency/high frequency) ratio is more controversial in its interpretations: in few studies it has been adopted as an index of physiological arousal and it could be interpreted as an indicator of sympathovagal equilibrium, yet this perspective is not fully accepted.^1^

On the contrary the speech does not show the same immediate modulation effects than singing. In past studies investigating the differential effect of the singing and speaking maternal interventions, we found that speech was particularly effective in inducing active waking states, while singing tended to maintain the same initial state in newborns.^49^ Moreover, speech directed to preterms tended to be more effective than singing in protecting newborns from pain and in increasing the oxytocin release during a heel prick procedure.^32^

Taken together, the overmentioned results confirmed the hypothesis that, at the origins of human connectedness, infant-directed singing and speech seem to have different functions in the co-regulatory dyadic processes. Singing to infants is a cross-cultural universal practice, expressed in different musical forms, but sharing common musical elements, such slower tempo, increased energy at lower frequencies and lengthened inter-phrase pauses when compared to adult-directed singing.^50,51^ When compared to infant-directed speech it shows a more repetitive and predictable structure, as it must respect a predefined melody and rhythmical structure. If infant-directed speech has been shown to be effective especially for developing improving infant’s learning^52^ (i.e. linguistic learning^53^) singing has predominantly, but not solely, the function to attract infants’ preferences and to maintain their attention,^54^ and it modulates infant’s levels of arousal.^55^

This important modulatory effect of the parental singing during development, which translates into the common practice of lullabies and play songs, needs to be investigated at a brain and physiological levels in future research. Neural correlates of speech perception in newborns have been largely investigated,^56^ but the modulatory effect of infant-directed singing, as a form of neural and physiological entrainment, could open new insights in the investigation of early co-regulation and synchrony between human beings.

Lastly, the same differences reported between the pre, during, and post maternal singing conditions were not evident in the control condition, in which the mothers were present but just watched the infant’s behavior. As a result, the mothers’ quiet presence next to the incubator is insufficient to control the infant’s cardiac responses and boost its vagal activity.

One of the major limitations of the present study is the lack of precise information on the language environment of the preterm infants in the NICUs. In several studies, the LENA device has been largely used as an important tool for characterizing the amount and the quality of the adult speech surrounding preterm newborns during the hospitalization period.^57^ However, in the present study the specific RCT design, with an active control, could minimize the effects of this missing information. As reported in the population description, the amount of time that parents spent in the NICU and the frequency of skin-to-skin contact were not different in the two groups. However, the intervention could have increased the total amount of time that parents spent in the two weeks in vocally communicating with their newborn. Unfortunately, this information is missing in the present study. Moreover, only deep sleep and crying states were coded by a trained nurse, and the intervention could begin only in the other states, that were not differentiated, and this is a limitation, as the newborn’s state can have an impact on his/her HRV components.

Another limitation of the study is the reduced sample size. Knowing that the recruitment was challenging and that the attrition rate in physiological signals tend to be elevated,^58^ we decided to increase the number of recordings for each patient, and to collect the initial baseline for each newborn, to strengthen the individualized measure. Finally, data on maternal stress state et the moment of the intervention is missing. Future research should collect contextual data on the families involved, to have a precise picture of the potential recruitment biases.

## Conclusion

Infant-directed singing modulates the newborns HRV, both as an immediate and as a cumulative effect, sustaining the ANS development in premature infants. The early maturation of the ANS and an activation of the parasympathetic system, compromised by the preterm birth, can allow in the long-term an earlier establishment of caring interactions and emotional connections between preterms and their parents, which in turn may have long-term benefits on the preterm infant’s development. Future analyses will correlate the physiological data with the degree of stress that parents perceived before and after the intervention and with long-term linguistic assessment.

### Supplementary information

Supplementary material

## Data Availability

All data are available upon request.
